# Ideas for addressing electronic harassment among adolescents attending a video blogging convention

**DOI:** 10.1186/s12889-018-5907-6

**Published:** 2018-08-06

**Authors:** Ellen Selkie, Yolanda Evans, Adrienne Ton, Nikita Midamba, Megan A. Moreno

**Affiliations:** 1 University of Michigan, Ann Arbor, MI USA; 2 University of Washington, Seattle, WA USA; 3 Columbia University, New York, NY USA; 4 Seattle Children’s Research Institute, Seattle, WA USA; 5 University of Wisconsin, Madison, WI USA

**Keywords:** Cyberbullying, Electronic harassment, Adolescents, Qualitative

## Abstract

**Background:**

Electronic harassment affects 20–40% of adolescents and has been associated with significant negative outcomes including physical (ex. headache, abdominal pain), psychological (ex. depression, anxiety), and psychosocial (ex. school avoidance) problems. Evidence-based strategies to address electronic harassment are lacking, and few studies have incorporated adolescent input into intervention design. The purpose of this study was to use a novel data collection approach to determine perspectives on electronic harassment intervention and prevention from a targeted group of highly engaged adolescent technology users.

**Methods:**

We conducted a qualitative survey of a purposeful sample of adolescents age 14 to 18 who were attending a video blogger convention in Seattle, Washington. Participants were approached by research staff and asked to read a vignette about an adolescent target of electronic harassment, then write down ideas for helping the target and/or preventing the scenario. Written responses were analyzed using a thematic analysis approach with an iterative comparative method to resolve any code discrepancies. We subsequently categorized codes into thematic code families to reach consensus about significant themes.

**Results:**

67 eligible adolescents completed the survey. 91% of participants were female with a mean age of 15.3 years (SD = 1.3). Code families emerged regarding people who could be involved in responses to electronic harassment: (1) Individuals targeted by electronic harassment, (2) Friends and bystanders, (3) Adults, and (4) Social media websites and policymakers.

**Conclusions:**

Findings demonstrate adolescent technology users’ views on several creative strategies to prevent or intervene with electronic harassment. These strategies can be categorized using a socioecological framework, demonstrating potential to address electronic harassment on multiple levels. Many suggested responses involved the target of electronic harassment, rather than the perpetrator; future education efforts may require additional focus on perpetrators for more upstream prevention.

## Background

Electronic harassment is a broad term referring to aggressive interpersonal interactions occurring online or through mobile devices and can be one component of adolescent bullying when it is repetitive and involves a power dynamic (i.e., cyberbullying) [1, 2]. In a review of electronic harassment studies, up to 26% of participants identified as perpetrators and over 42% as targets [3]. Other studies have found females to be more likely to be affected than males [4–6]. Electronic harassment is associated with physical and mental health concerns, including insomnia, recurrent abdominal pain, anxiety, depression, substance use, and suicidal ideation [3, 7–10]. While electronic harassment often co-occurs with in-person harassment or bullying, it has distinct characteristics such as potential anonymity for perpetrators, a widespread audience, and persistence outside of school hours leading some researchers to speculate that electronic harassment could be more harmful than other forms of aggression [11, 12]. As such, mitigation efforts regarding electronic harassment may require different approaches from traditional interventions.

School programs for prevention of non-electronic harassment have shown positive effects on reducing in-person behaviors, though few have been studied with regard to effects on electronic harassment. Programs that have been evaluated for impact on electronic harassment have shown mixed results [13–15]. For example, a longitudinal study in Australia found that a school program focused on online safety was associated with decreased rates of both perpetration of and victimization from electronic harassment [16]. On the other hand, a study in Finland found electronic harassment rates increased when middle school students perceived that their teachers could stop in-person bullying [17]. A study in the United States found that some high school teachers may not be receptive to electronic harassment prevention programs [18]. These findings suggest that traditional interventions may not address harassment that occurs in an online environment, especially if it can happen outside of school hours.

Interventions specifically directed at electronic harassment have been increasing in number but have shown mixed results [19]. One popular option is to hold an assembly or watch a video to discuss electronic harassment; however, evidence of long-term impact is lacking [20, 21]. One pilot study showed that a longer online curriculum reduced electronic harassment intent, but did not change attitudes [22]. Other research has explored technical solutions such as blocking bullies on social media, but the effect of these behaviors on electronic harassment rates has not been evaluated [23]. Interventions that have shown promise in reducing electronic harassment involve multi-session education programs utilizing both adult and adolescent leadership [16, 24].

Adolescents are avid consumers of social media and mobile technology, data from a Pew Research Center survey showed 89% of adolescents reported going online multiple times a day. Most adolescents use multiple platforms daily, including YouTube (85%), Instagram (72%), Snapchat (69%), Facebook (51%), and Twitter (32%). Internet and mobile use as well as the prevalence of electronic harassment in adolescent online communities establish adolescents as key stakeholders in developing electronic harassment interventions. Qualitative approaches to adolescent perspectives on the topic of electronic harassment are growing, and various methodologies exploring adolescent generated solutions have been utilized [25, 26]. Some studies have approached this topic using surveys that asked participants to choose from predetermined solutions [27, 28]. One study used participatory research to explore intervention ideas with Dutch elementary schools, and another used open-ended questions to elicit coping strategies in Swedish adolescents [29, 30]. This study aims to add to the existing body of participatory research by engaging adolescents in the sociocultural context of the United States to answer the following questions: Who do adolescents think should address the problem of electronic harassment? What are the specific strategies that adolescents suggest for prevention of electronic harassment? Therefore, the purpose of this study was to use a novel data collection approach to determine perspectives on electronic harassment intervention and prevention from a group of highly engaged adolescent technology users.

## Methods

### Setting

Data collection took place on a single day in summer 2014 at VloggerFair, an annual convention for video bloggers (vloggers) to network and discuss vlogging. This event was held in Seattle, WA, and had over 2000 attendees, mostly female (70%), and on average 19 years old [31]. VloggerFair attracts a largely adolescent audience, organizers attribute this to the widespread adoption of YouTube (the most popular video sharing website) as a form of entertainment, education, and information, as well as the presence of prominent vloggers [31]. For example, many female adolescents attended VloggerFair to see Tyler Oakley, who has nearly eight million followers on YouTube [32].

The VloggerFair organizers provided permission to conduct research at the fair. In discussions with the event planning committee, we learned that many adolescents attend with groups of friends rather than their parents. In order to increase our ability to capture data from as many adolescent attendees as possible, the Western Internal Review Board approved a waiver of parental consent.

### Participants and recruitment

**Fig. 1:**
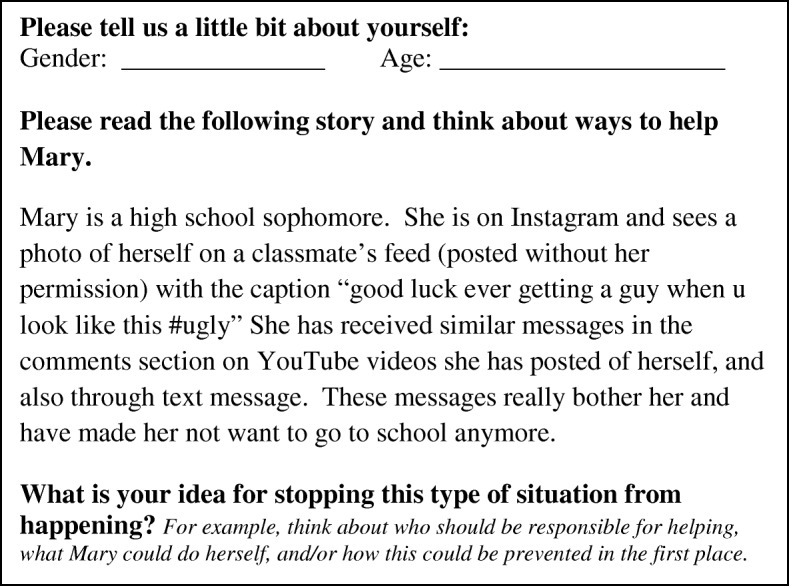
Electronic harassment scenario and survey

Participants for this study included adolescents between the ages of 14 and 18 attending VloggerFair. Study data were collected over a 4 h period during the convention. A team of four investigators—two Adolescent Medicine physicians (Y.E. and E.S.) and two research assistants (A.T. and N.M.)—attended VloggerFair, where they approached attendees in line for booths and asked if they would be interested in participating in a short research study. After screening for age eligibility, recruiters obtained consent to participate in the study. Participants were then handed a paper form with a vignette describing a typical electronic harassment scenario (Fig. 1) and questions to complete while standing in line. Surveys took between one and ten minutes to complete. The surveys were anonymous and kept separate from consent information. After completing the survey, participants were given the option to enter their contact information to enter a drawing for a $15 gift card. Contact information was kept separate from surveys and consent forms and was destroyed after the gift card drawing.

### Measures

This study employed an open-ended qualitative survey format in which, after completing demographic questions, participants read a vignette about an adolescent girl who was being cyberbullied (Fig. 1). The vignette was written by an Adolescent Medicine physician (E.S.) based on aggregated clinical experiences with patients who had been targets of electronic harassment as well as definitions of electronic harassment (i.e., deliberately hurtful, using electronic devices, and associated with harm) [33]. All research team members reviewed the vignette prior to use in the survey. Research has shown that both adolescents and researchers have differing definitions of cyberbullying [25, 34]. As such, the term “cyberbullying” was intentionally excluded from the vignette to capture responses that considered broad electronic harassment behavior. After reading the vignette, participants were asked to write interventions and/or prevention strategies to address the electronic harassment behavior (Fig. 1).

### Analysis

Four investigators (E.S., Y.E., A.T., N.M.) used a thematic approach to analyze the data. All investigators had previously participated in qualitative research projects, ensuring familiarity with analysis principles. Investigators initially reviewed survey responses individually to extract relevant quotations from each response and identify repeated aspects of those quotations, thus creating a preliminary scheme of codes. The investigators then met to discuss and achieve consensus on code categories. Specifically, representative quotations for each code were selected by individual investigators, and were then reviewed by the group to achieve consensus and create a final codebook. Investigators then returned to the data and coded it using these categories obtained through consensus. Group participation in discussion and revision of the codebook allowed us to achieve internal validity. After coding using the designated categories, codes were sorted into code families. Themes were derived from the most prevalent and salient codes [35]. By the end of coding, all 4 investigators were in agreement that within the sample, theoretical saturation of patterns and themes had been reached.

## Results

### Demographics

A total of 67 adolescents completed surveys. Participants were 91% female with a mean age of 15.3 years (SD 1.3 years). The mean number of words per response was 34 (SD = 18 words, range = 8–80 words).

### Responses

Participants identified four general groups that could be involved when electronic harassment occurs: the individual victim, their peers, adults in the victim’s life, and organizations such as social media websites and legal systems.

### Individual interventions

#### Ways to respond

Participants identified several ways that individuals could approach an electronic harassment situation. For example, ignoring hurtful messages was a suggested strategy: “Don’t let the haters get to you;” “[the victim] should not message back and not give the bully any attention.” Some participants recommended engaging or standing up to the person perpetrating the harassment: “Kill them with kindness. Stick up for yourself.” “If she thinks she can handle the situation on her own then she can try to confront the people leaving cruel comments.”

#### Cognitive restructuring

Several participants described ways that the victim of harassment could cope through reframing the situation. For example, the victim could focus on positivity toward herself and others: “know in her head that she shouldn’t care what others think, she is beautiful;” “I feel like if it’s only one person [bullying], I bet there’s tons of other people who wouldn’t say anything like that. Think about all the people who think the opposite of this one person.” They also stated that a victim could cope through internally invalidating a perpetrator, “If they are trying to pull you down, it means you’re already above them;” “The haters are just jealous.”

#### Technical strategies

Participants had several suggestions for using technology itself to help with electronic harassment. An individual victim could take action by blocking a user who posted negative content or reporting the user to the website: “Block the people that are doing it and report it.” “Report whoever posted these videos to the school or online.” In addition, participants recommended making profiles private on social media and disabling comments as prevention mechanisms: “[the target] should make her account PRIVATE so that those not wanted on her page can’t take her picture.” Finally, multiple participants recommended a hiatus from social media, “Get off the social media. Go and find people who support you for who you are…” and “I think she should take a break from social media and then talk to a parent or teacher. Then be careful with other social media sites.” in the face of electronic harassment.

### Peer interventions

#### Reaching out to the victim

Participants acknowledged the importance of peer support, noting that friends should support a victim of electronic harassment by proactively giving her positive comments online or in person. “Make her feel good by giving her lots of compliments all the time and making sure she knows that she’s always got someone she can go to for anything.” “Everyone has haters and people who knock you down, but everyone has people who support them as well. Focus on the people who support you.”

#### Responding to the perpetrator

While reaching out to the victim was deemed important, participants also endorsed engaging with the perpetrator of harassment, standing up for their peer and assisting them in finding help. “…get support from her friends first…Her fans should step up and stand up for her on YouTube and her friends should do the same.”

### Adult interventions

#### Trusted adults

Support for the victim of electronic harassment was mentioned as an important component of intervention. In particular, the word “trusted” was repeatedly used to describe these adults, including parents, teachers, and school counselors. “Tell a trusted adult, talk to someone about it.” “Talk to people of authority like parents or someone you trust…” “She should talk to an adult about it like a counselor.” “Ignore it, talk to parents, teachers, close friends.”

### Systems interventions

#### Schools

Schools were mentioned as sources of preventive policies and interventions at the systems level. One participant noted difficulty in expecting the school intervene in incidents occurring online: “sometimes the school district doesn’t have specific rules for the Internet, so she would need to be persistent.” Involving the school disciplinary system was described as being especially necessary if electronic harassment was continuing despite other efforts by the individual. “[the perpetrator] should receive discipline from the school.” School programming against electronic harassment was also mentioned: “This could be prevented by a bullying awareness class/panel in school people HAVE to learn about.”

#### Legal systems

Looking to an even broader level of intervention, participants noted the importance of policy and law enforcement in addressing electronic harassment. Participants often suggested the victim should show police the quotes or texts related to electronic harassment, particularly in severe cases. “First off report the picture that is on Instagram. Don’t delete the texts and comments because if the police need to get involved, there is evidence.” “…if it starts getting obsessive then she should contact authorities. sadly telling people to stop doesn’t always work.” “Mary could talk to her parents friends teachers and if really serious the police.” “She should show the police or her parents the texts.” Participants also emphasized the need for laws regarding electronic harassment and cyberbullying. “I think cyber bullying should be illegal everywhere not just in some states and the person who posted it should be charged with assault and it’s not fair to the kids who have been bullied…”.

#### Websites

Finally, social media websites such as Instagram and YouTube were cited as proposed sources of help for electronic harassment intervention and prevention: “I think Instagram should delete [electronic harassment posts]. Posts should be reviewed whether or not it was reported or not.” “The social media sites should block images or users that are being mean to other people.” “Social media websites are responsible and should have a way to get these photos deleted if they negatively impact someone’s life.”

## Discussion

**Fig. 2:**
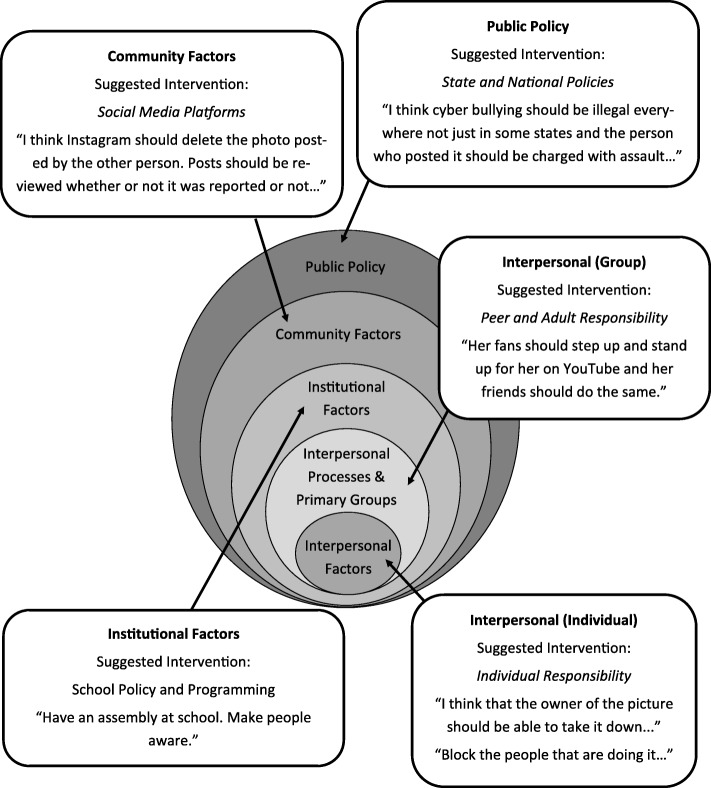
Social ecological model with key quotes

VloggerFair participants had a variety of ideas for confronting electronic harassment at multiple levels of social structure including the individual, peer, adult/authority, and systems levels. These groups may be understood within the framework of the Socioecological Model (SEM) [36, 37]. The SEM proposes five sources of influence on health behaviors: intrapersonal factors, interpersonal processes and primary groups, institutional factors, community factors, and public policy. These levels of influence provide an analytic framework for understanding health behavior and identifying interventions [36, 37]. The model has been used to examine bullying and harassment along with other areas of adolescent health behavior, including nutrition, physical activity, and sexual health, in a social ecological framework [38–41]. While our study design was not specifically guided by the SEM, we realized during post-hoc analysis that the SEM’s proposed sources of influences on behaviors were represented in the comments provided by the VloggerFair attendees (Fig. 2).

In their proposed interventions, at the individual level most participants placed the burden of addressing electronic harassment on targets, rather than perpetrators. This may be partially due to the scenario presented, which was narrated in the third person from the perspective of a target. Adolescents may also feel that while they cannot control the behavior of perpetrators, it may feel more realistic for targets to have their own coping strategies. One concern with this is the possible perception of electronic harassment as a normal or expected part of daily life. Given that normative beliefs about electronic harassment have been shown to contribute to increased perpetration of electronic harassment, further research is needed to determine whether primary prevention of electronic harassment can be viewed as feasible among adolescents [42, 43].

Participants in our study valued the bystander as an effective electronic harassment intervention. In in-person bullying and harassment situations, the proportion of bystanders who actually respond is quite low [44–46]. However, bystanders in electronic harassment may be more likely to intervene (e.g. telling the victim they are sorry the incident happened) but may also contribute negatively to the aggression by spreading hurtful content or encouraging the perpetrator further [47]. A focus group study exploring bystander involvement in adolescents found that poor social acceptability, low self-efficacy, and pessimism of a positive result were stated as reasons for not intervening as a bystander [48]. Further research should continue to assess barriers to bystander intervention and evaluate strategies to empower bystanders in a virtual setting.

Participants endorsed telling trusted adults and school officials about electronic harassment. This is in contrast to other studies, which have found that youth fear telling an adult due to potential loss of technology privileges [27, 29]. Furthermore, other research found students perceived teachers as ineffective at stopping electronic harassment [49]. The responses to our survey may represent changing opinions among adolescents about the value of adult support in electronic harassment situations.

Finally, participants suggested systems change as a way to address electronic harassment. Few precedents have been set to guide legal policy in this area. State laws requiring schools to enact anti-bullying policies have shown promise in reducing non-electronic and electronic harassment rates, but when electronic harassment occurs outside of the school setting, less is known about the effectiveness of such policies [50]. Furthermore, responses to electronic harassment on the part of social media websites have not been empirically studied with regard to prevention of further incidents. Currently, few incentives exist for the social media industry to implement rigorous measures against electronic harassment other than the concepts of social responsibility and general citizenship, which may be outweighed by financial gain [51]. Nonetheless, a 2016 National Academies Report on Bullying made a specific recommendation to social media industry to consider systems to track and respond to electronic harassment [52]. Research and partnership with industry experts could elucidate other barriers to addressing electronic harassment at the level of online administration.

Our study represents a novel method for obtaining qualitative data from adolescents actively engaged in digital technology. Obtaining data at events with high proportions of adolescent attendees is feasible, and can provide rich data if a study’s research question is related to the focus of the event. However, this data collection approach is not without limitations. First, the sample consisted of attendees of a single event on a single day, making triangulation of data from multiple sources impossible. This was also a public event with thousands of attendees moving between booths and speakers, and since we had research staff in various locations throughout the fair, we were unable to accurately quantify response rate. Participants were approached by research staff who were older than the participants, which may have affected willingness to participate in the study. The short length of the survey and the distractions of the VloggerFair venue may have led to participants answering questions in less depth than they might have in a more focused setting. Indeed, some participant responses were reflective of common messaging given in school assemblies about electronic harassment, for example, “tell a trusted adult.” This contrast to previously observed lack of reporting to adults (as discussed above) suggests a rote response that adults have told adolescents they “should” give. Social acceptability bias may have similarly influenced participant responses given that many participants were at the event with peers. Future work could expand the length of survey for more in-depth analysis.

The sample was also largely female (reflecting the female predominance among those attending VloggerFair), and the survey’s vignette involved a female target. Perspectives of male respondents were therefore underrepresented, and results may have been different if a male target was the subject of the vignette. However, since females are more likely to be involved in electronic harassment [4, 5], this study was well-positioned to capture the opinions of this group. Further research using a larger sample with more balanced gender representation will be important for intervention design.

We did not ask participants whether they had experienced electronic harassment personally, as we avoided asking for personal information given the waiver of parental consent. As such, we are unable to characterize how suggestions for intervention and prevention vary with experience with electronic harassment. This would be an important area for future study. However, the event was one attracting adolescents with high technology use, which has been associated with increased rates of electronic harassment perpetration and victimization [53]. Thus, our data represent the opinions of the population most invested in electronic harassment.

## Conclusion

We learned that adolescents see electronic harassment as a multifaceted problem with opportunities for intervention in several ecological domains. Public campaigns, grassroots efforts, and individual counseling can empower adolescents (targets and bystanders) to stand up to perpetrators, and raise awareness of the responsibilities adults have to support youth. At the institutional level, schools must include electronic harassment in curricula to teach responsible technology use and interpersonal skills. Schools must also adopt and enforce electronic harassment policies. At the community level, social media companies should promote positive messaging and diminish negative messaging, though, for example, algorithmic moderation of their platforms. Finally, updated state and national harassment policies are needed to reflect the growing use of online platforms, to raise awareness for prevention, and define developmentally appropriate consequences for perpetrators. Future intervention design should utilize the SEM framework and include youth voices to maximize acceptance and application as well as benefit for this population.

## Abbreviations

SEM: Socio-ecological model
vlogger: Video blogger

## References

[1] Smith PK (2008). Cyberbullying: its nature and impact in secondary school pupils. J Child Psychol Psychiatry.

[2] Olweus D (1993). Bullying at school: what we know and what we can do. Understanding children’s worlds.

[3] Hamm MP (2015). Prevalence and effect of Cyberbullying on children and young people: a scoping review of social media studies. JAMA Pediatr.

[4] Beckman L, Hagquist C, Hellstrom L (2013). Discrepant gender patterns for cyberbullying and traditional bullying - an analysis of Swedish adolescent data. Comput Hum Behav.

[5] Connell NM (2014). Badgrlz? Exploring sex differences in cyberbullying behaviors. Youth Violence Juvenile Justice.

[6] Li Q (2006). Cyberbullying in schools: a research of gender differences. Sch Psychol Int.

[7] Kowalski RM, Limber SP (2013). Psychological, physical, and academic correlates of cyberbullying and traditional bullying. J Adolesc Health.

[8] Hinduja S, Patchin JW (2010). Bullying, cyberbullying, and suicide. Arch Suicide Res.

[9] Sourander A (2010). Psychosocial risk factors associated with cyberbullying among adolescents: a population-based study. Arch Gen Psychiatry.

[10] Aboujaoude E (2015). Cyberbullying: review of an old problem gone viral. J Adolesc Health.

[11] Sticca F, Perren S (2013). Is cyberbullying worse than traditional bullying? Examining the differential roles of medium, publicity, and anonymity for the perceived severity of bullying. J Youth Adolesc.

[12] van Geel M, Vedder P, Tanilon J (2014). Relationship between peer victimization, cyberbullying, and suicide in children and adolescents: a meta-analysis. JAMA Pediatr.

[13] Mishna F (2011). Interventions to prevent and reduce cyber abuse of youth: a systematic review. Res Soc Work Pract.

[14] Olweus D (2012). Cyberbullying: an overrated phenomenon?. European J Developmental Psychology.

[15] Williford A (2013). Effects of the kiva Antibullying program on Cyberbullying and Cybervictimization frequency among Finnish youth. J Clin Child Adolesc Psychol.

[16] Cross D (2016). Longitudinal impact of the cyber friendly schools program on adolescents’ cyberbullying behavior. Aggress Behav.

[17] Christian Elledge L (2013). Individual and contextual predictors of cyberbullying: the influence of children's provictim attitudes and teachers’ ability to intervene. J Youth Adolesc.

[18] Stauffer S (2012). High school teachers’ perceptions of cyberbullying prevention and intervention strategies. Psychol Sch.

[19] Hutson E, Kelly S, Militello LK (2018). Systematic review of Cyberbullying interventions for youth and parents with implications for evidence-based practice. Worldviews Evid-Based Nurs.

[20] Roberto AJ (2014). Outcome evaluation results of school-based Cybersafety promotion and Cyberbullying prevention intervention for middle school students. Health Commun.

[21] Roberto A (2017). The short-term effects of a Cyberbullying prevention intervention for parents of middle school students. Int J Environ Res Public Health.

[22] Lee MS (2013). Cyber bullying prevention: intervention in Taiwan. PLoS One.

[23] Cowie H (2011). Coping with the emotional impact of bullying and cyberbullying: how research can inform practice. Int J Emot Educ.

[24] Palladino BE, Nocentini A, Menesini E (2016). Evidence-based intervention against bullying and cyberbullying: evaluation of the NoTrap! Program in two independent trials. Aggress Behav.

[25] Navarro R, Serna C (2016). Spanish Youth Perceptions About Cyberbullying: Qualitative Research into Understanding Cyberbullying and the Role That Parents Play in Its Solution.

[26] Wang CW, et al. A Qualitative Study on Cyberbullying and Traditional Bullying among Taiwanese High School Students [abstract]. In: Proceedings of the 19th International Conference on Behavioral Medicine. Stockholm; 2017.

[27] Cassidy W, Jackson M, Brown KN (2009). Sticks and stones can break my bones, but how can pixels hurt me?: students’ experiences with cyber-bullying. Sch Psychol Int.

[28] Monks CP, Robinson S, Worlidge P (2012). The emergence of cyberbullying: a survey of primary school pupils’ perceptions and experiences. Sch Psychol Int.

[29] Baas N, de Jong MD, Drossaert CH (2013). Children’s perspectives on cyberbullying: insights based on participatory research. Cyberpsychol Behav Soc Netw.

[30] Frisen A, Berne S, Marin L (2014). Swedish pupils’ suggested coping strategies if cyberbullied: differences related to age and gender. Scand J Psychol.

[31] LockerGnome. *VloggerFair 2014 Media Kit*. 2014 May 12, 2016]; Available from: https://web.archive.org/web/20140313111951/http://mediakit.vloggerfair.com/audience.

[32] Tyler Oakley. YouTube. [cited 2016 January 27]; Available from: https://www.youtube.com/user/tyleroakley.

[33] Patchin JW, Hinduja S (2006). Bullies move beyond the schoolyard a preliminary look at cyberbullying. Youth Violence Juvenile Justice.

[34] Selkie EM, Fales JL, Moreno MA (2016). Cyberbullying prevalence among US middle and high school-aged adolescents: a systematic review and quality assessment. J Adolesc Health.

[35] Braun V, Clarke V (2006). Using thematic analysis in psychology. Qual Res Psychol.

[36] McLeroy KR (1988). An ecological perspective on health promotion programs. Health Educ Behav.

[37] Bronfenbrenner U (1977). Toward an experimental ecology of human development. Am Psychol.

[38] Espelage DL (2014). Ecological theory: preventing youth bullying, aggression, and victimization. Theory Pract.

[39] Hong JS (2016). Understanding the correlates of face-to-face and cyberbullying victimization among U.S. adolescents: a social-ecological analysis. Violence & Victims.

[40] Svanemyr J (2015). Creating an Enabling Environment for Adolescent Sexual and Reproductive Health: A Framework and Promising Approaches. J Adolescent Health.

[41] Baskin ML (2015). Social and cultural environment factors influencing physical activity among African-American adolescents. J Adolesc Health.

[42] Ang RP, Tan KA, Talib Mansor A (2011). Normative beliefs about aggression as a mediator of narcissistic exploitativeness and cyberbullying. J Interpers Violence.

[43] Heirman W, Walrave M (2012). Predicting adolescent perpetration in cyberbullying: an application of the theory of planned behavior. Psicothema.

[44] Nishina A, Bellmore A (2010). When might peer aggression, victimization, and conflict have its largest impact? Microcontextual considerations. The Journal of Early Adolescence.

[45] Lynn Hawkins D, Pepler DJ, Craig WM (2001). Naturalistic observations of peer interventions in bullying. Soc Dev.

[46] O'Connell P, Pepler D, Craig W (1999). Peer involvement in bullying: insights and challenges for intervention. J Adolesc.

[47] Jones LM, Mitchell KJ, Turner HA (2015). Victim reports of bystander reactions to in-person and online peer harassment: a national survey of adolescents. Journal of youth and adolescence.

[48] Desmet A (2012). Mobilizing bystanders of cyberbullying: an exploratory study into behavioural determinants of defending the victim. Stud Health Technol Inform.

[49] Tangen D, Campbell M (2010). Cyberbullying prevention: one primary school’s approach. Aust J Guid Couns.

[50] Hatzenbuehler ML (2015). Associations between antibullying policies and bullying in 25 states. JAMA Pediatr.

[51] Youmans WL, York JC (2012). Social media and the activist toolkit: user agreements, corporate interests, and the information infrastructure of modern social movements. J Commun.

[52] National Academies of Sciences, E.a.M (2016). Preventing Bullying Science, Policy, and Practice.

[53] Rice E (2015). Cyberbullying perpetration and victimization among middle-school students. Am J Public Health.

